# New molecular evidence of the genus *Hydrurus* (Chrysophyceae) and descriptions of *Hydrurusfoetidus* (Villars) Trevisan on the basis of morphology and phylogeny

**DOI:** 10.3897/BDJ.12.e137389

**Published:** 2024-11-01

**Authors:** Junxue Hao, Yalu An, Fangru Nan, Junping Lv, Qi Liu, Xudong Liu, Shulian Xie, Jia Feng

**Affiliations:** 1 Shanxi University, Taiyuan, China Shanxi University Taiyuan China

**Keywords:** China, Chrysophyceae, evolution, *Hydrurus*, morphology, molecular phylogeny

## Abstract

**Background:**

The genus *Hydrurus* contains a solitary species, *Hydrurusfoetidus*. Its thalli exhibit a remarkable structure, consisting of a firm central axis surrounded by peripheral branches, all enveloped within a viscous, gelatinous coating. Molecular data pertaining to the genus *Hydrurus* are scarce, necessitating further investigation into its phylogenetic relationships.

**New information:**

A new site with benthic freshwater alga *Hydrurusfoetidus* (Villars) Trevisan has been discovered in the Fenhe River in Shanxi Province, China. The physical and chemical parameters of water were meticulously measured and documented. Detailed morphological observations were conducted on the specimen, measuring different cell categories. The SSU, LSU, ITS and *rbc*L DNA sequence data of *H.foetidus* collected from Shanxi were determined. An extensive three-gene phylogenetic tree was constructed, revealing a strong relationship between the specimen in this study and *H.foetidus* specimen from Norway. Time-calibrated molecular phylogenetic analysis further indicated that the genus *Hydrurus* diverged approximately 125 million years ago (Early Cretaceous), while the two *H.foetidus* strains from Shanxi, China and Norway diverged approximately 6 million years ago (Neogene). The results of this study supplement new molecular evidence for *H.foetidus* and contribute significantly to our understanding of the geographical distribution and evolutionary history of the genus *Hydrurus*.

## Introduction

*Hydrurus* C. Agardh, the only genus of Hydruraceae, was established in 1824 (Agardh 1824). It contains only one species, *H.foetidus* (Villars) Trevisan (Wei 2018). *Hydrurusfoetidus* exhibits a macroscopic and benthic nature, which differs from other golden algae (Rott et al. 2006a, Klaveness et al. 2011). *Hydrurus* is widely distributed worldwide, especially in the Northern Hemisphere (Kristiansen and Preisig 2011, Klaveness 2017, Klaveness 2019). As a typical cold-water species, *H.foetidus* can be found in cold mountain streams and lowland rivers during early spring and winter, as well as in rivers with consistently low temperatures throughout summer or autumn (Ward 1994, Rott et al. 2006b, Kristiansen and Preisig 2011, Klaveness 2017, Klaveness 2019). During winter, a noteworthy phenomenon arises when the psychrophilic chrysophyte, *H.foetidus*, causes blooms in melting snow (Remias et al. 2013, Lutz et al. 2018, Remias et al. 2020).

*H.foetidus* typically attaches to stones and forms bushy thalli on riverbed materials (Kristiansen and Preisig 2011). *Hydrurus* is often sensitive to the environment and temperature, light and carbon dioxide are key factors affecting its growth (Bursa 1934, Kawecka 2003, Nozaki et al. 2020). Notably, the optimal temperature range for the growth of *Hydrurus* has been reported to be 2–12℃ (Klaveness 2019). Although algae are multicellular, single cells within the thalli can slide in polysaccharide tubes or be released (Klaveness et al. 2011, Bråte et al. 2019). In addition, each cell typically possesses multiple contractive vacuoles (Klaveness 2019). Furthermore, *Hydrurus* holds immense potential as a rich food source for fungi, protists and insect larvae, providing valuable nutrients, such as polyunsaturated fatty acids and polysaccharides (Milner et al. 2001, Zah et al. 2001, Milner et al. 2009, Klaveness 2017).

Phylogenetic studies of the genus *Hydrurus* have been limited by a lack of molecular evidence. Only three sequences (one of 5S rRNA, one of 18S rRNA and another of 28S rRNA) are available via the National Centre for Biotechnology Information (NCBI) GenBank database (Lim et al. 1986, Klaveness et al. 2011). Although Bråte et al. (2019) proposed an extensive next-generation sequencing dataset, they did not delve into the analysis of genome sequences; thus, the analysis of genome sequences remains unexplored. Molecular clock analyses and specific molecular markers, such as ITS and *rbc*L, have been employed in early chrysophyte studies (Jo et al. 2013, Siver et al. 2015, Jo et al. 2016, Čertnerová et al. 2019, Škaloud et al. 2020). However, these methods have yet to be widely applied in the genus *Hydrurus*. Additionally, the absence of molecular sequences and limited fossil records hinder a clear understanding of the position and evolutionary history of the genus *Hydrurus* within the Chrysophyceae. To accurately determine the phylogenetic position of *Hydrurus*, we need more sequence data and explore additional molecular markers.

In this study, we conducted time-calibrated molecular phylogeny, based on concatenated SSU, LSU and *rbc*L rDNA sequences to investigate the classification and evolution of the genus *Hydrurus*. This study aimed to:

1. describe *H.foetidus* collected from China, based on morphological characteristics;
2. provide new molecular data for *H.foetidus* and infer the phylogenetic relationships amongst chrysophyte species;
3. comprehend the species diversity and infer the divergence time of *Hydrurus* species;
4. contribute to the geographical distribution of *Hydrurus* and enhance biodiversity records of freshwater chrysophytes.

## Materials and methods

### Sample collection

**Figure 1.**
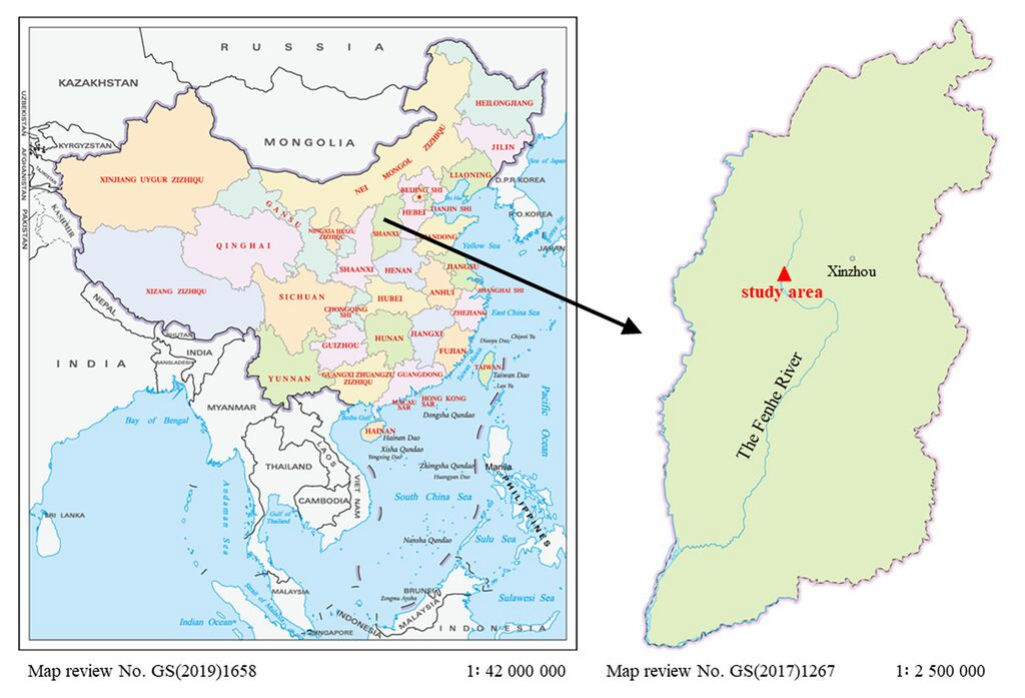
Map of the study area.

The materials were collected from the Shanxi Province of China in March 2023 (Fig. 1). The materials were directly picked up from stones using knives and tweezers and subsequently transferred to the laboratory. Water quality parameters, including water temperature (WT), pH, salinity, Secchi depth (SD), dissolved oxygen (DO), electronic conductivity (EC) and total dissolved solids (TDS), were measured using hand-held meters (YSI Professional Plus Multiparameter Water Quality Instrument 19E102487, YSI Incorporated, Brannum Lane Yellow Springs, Ohio, USA). COD, ammonium (NH4^+^), total nitrogen (TN) and total phosphorus (TP) were determined by the dichromate method, Nessler’s reagent spectrophotometry, ultraviolet spectrophotometry and ammonium molybdate spectrophotometric, respectively (State Environmental Protection Administration 2002). The samples were washed with sterile water several times to remove impurities. Voucher specimens were preserved in 4% formaldehyde. Voucher specimens were deposited in the Herbarium of Shanxi University (SXU), Shanxi University, Taiyuan, Shanxi Province, China (Voucher number: SXU-SX230328-31).

### Morphological observations

Morphological characters of the specimens were observed under an Olympus BX-51 microscope (Olympus, Tokyo, Japan), equipped with a digital camera for photographing (DP72 Olympus, Tokyo, Japan).

### DNA extraction, amplification and sequencing

Total DNA was extracted from the fresh thalli collected from Shanxi Province using a plant DNA extraction kit (Sangon Biotech, Shanghai, China). The four genes (SSU, LSU, ITS and *rbc*L) were amplified using the paired primers listed in Table 1. The polymerase chain reaction (PCR) amplifications were conducted in 50 μl volumes containing 37.75 μl ddH_2_O, 5.0 μl 10× buffer, 4.0 μl 2.5 mM dNTPs, 0.25 μl Taq DNA polymerase (Sangon Biotech, Shanghai, China), 1.0 μl of each primer (10 mM) and 1.0 μl of genomic DNA. The amplifications were performed using the following programmes: 94℃ for 5 min, 35 cycles of 94℃ for 30–60 s, 46.5–59℃ for 30–60 s and 72℃ for 2 min and final 72℃ for 10 min. The reaction was undertaken in a MyCycler thermal cycler (Bio-Rad, Hercules, CA, USA). The sequencing was performed on an ABI 3730XL sequencer. The DNA sequences generated in this study have been deposited in GenBank under accession numbers (OR230247, OR336050, OR284295 and PP025381).

### Sequence alignment and phylogenetic analysis

Newly-obtained sequences in this study and downloaded sequence data from GenBank (listed in Suppl. material 1) were aligned using MAFFT version 7 (Katoh et al. 2019). The sequences of SSU, LSU and *rbc*L were concatenated, based on the methods of Zhang et al. (2020). Pairwise genetic P-distances of concatenated sequences were calculated in MEGA 5.0 (Tamura et al. 2011). *Synchromagrande* and *Nannochloropsislimnetica* were used as outgroups in the phylogenetic tree. The appropriate model was built using the software PartitionFinder 2, with all algorithm and AIC criteria (for BI: Subset (1)(2)(3) = GTR + I + G; for ML: Subset (1)(2)(3) = GTR + I + G) (Lanfear et al. 2017). IQ-TREE was used to construct Maximum Likelihood (ML) trees with 5000 ultrafast bootstraps repetitions (Nguyen et al. 2015). Bayesian Inference (BI) phylogenies were inferred using MrBayes 3.2.6 and the BI analysis was run for 3,000,000 generations (Ronquist et al. 2012). The resulting phylogenetic trees were edited using FigTree 1.4.2 (http://tree.bio.ed.ac.uk/software/figtree/). Adobe Illustrator CS5 (Adobe System, San Jose, CA, USA) was used to optimise the graphics of all trees.

### Molecular clock analyses

We employed a Bayesian Inference method with a relaxed clock model using BEAST version 2.6.6 (Bouckaert et al. 2014) to conduct phylogeny and simultaneously estimate branch divergence times. We used the uncorrelated lognormal model to estimate variation rates across all branches. We used fossil calibrations as probabilistic priors. The lognormal priors were used for spits between the species *Mallomonasdenticulata* and M.striatavar.serrata and between *M.elevata* and *M.foveata*. Both calibrations were based on an offset of 38 Ma, a mean of 0.5 Ma and a standard deviation of 1.0, which represents a minimal age estimate for the majority of fossils of *Mallomonas* species from the Giraffe Pipe locality (Creaser et al. 2004, Doria et al. 2011, Siver et al. 2015). A generalised time reversible (GTR) + gamma site model was applied to the three-gene concatenated dataset and a Yule tree prior was used as a speciation model. The analysis was run for 50 million generations with the chain sampled every 1000 generations. Convergence of parameter estimates and estimation of burn-in was checked using the programme Tracer version 1.7 (Rambaut et al. 2018). The initial 5,000,000 (10%) were removed and the rest were retained to construct the final chronogram with 95% posterior probabilities (PP) and age estimates for all nodes. The resulting phylogenetic trees were edited using FigTree 1.4.2 (http://tree.bio.ed.ac.uk/software/figtree/) and optimised using Adobe Illustrator CS5 (Adobe System, San Jose, CA, USA).

### ITS2 secondary structures

The ITS2 sequences of the genera *Mallomonas* and *Synura* were downloaded from GenBank and aligned with the sequence of *Hydrurusfoetidus* obtained in this study using MAFFT version 7 (Katoh et al. 2019). The ITS2 secondary structure of *H.foetidus* was constructed using the mfold computer programme (Zuker 2003) and VARNA (Darty et al. 2009).

## Data resources

All the sequences in this study were retrieved from GenBank.

## Taxon treatments

### Hydrurus foetidus

D4B9B93D-257A-5AF9-8693-2A73061D3081

https://www.ncbi.nlm.nih.gov/nuccore/?term=Hydrurus

#### Materials

- **Type status:** Other material. **Occurrence:** catalogNumber: SXU-SX230328; recordedBy: Liu En-Hui; occurrenceID: B584ADE9-8946-54F2-B493-004E12833296; **Taxon:** scientificName: *Hydrurusfoetidus*; **Location:** locality: the Fenhe River, Shanxi Rrov., China; verbatimElevation: 1869.2 m; verbatimCoordinates: 38.8574 N 112.0831 E; **Identification:** identifiedBy: Hao Jun-Xue; **Event:** year: 2023; month: 3; **Record Level:** type: specimen; language: en; collectionCode: Algae
- **Type status:** Other material. **Occurrence:** catalogNumber: SXU-SX230329; recordedBy: Liu En-Hui; occurrenceID: F1507A28-C087-5D3F-9053-42379E517331; **Taxon:** scientificName: *Hydrurusfoetidus*; **Location:** locality: the Fenhe River, Shanxi Rrov., China; verbatimElevation: 1506.89 m; verbatimCoordinates: 38.6605 N 112.1085 E; **Identification:** identifiedBy: Hao Jun-Xue; **Event:** year: 2023; month: 3; **Record Level:** type: specimen; language: en; collectionCode: Algae
- **Type status:** Other material. **Occurrence:** catalogNumber: SXU-SX230330; recordedBy: Liu En-Hui; occurrenceID: 860CA1A8-0FA5-58A3-B5BD-E9EDC98518C9; **Taxon:** scientificName: *Hydrurusfoetidus*; **Location:** locality: the Fenhe River, Shanxi Rrov., China; verbatimElevation: 1497.4 m; verbatimCoordinates: 38.5541 N 112.0191 E; **Identification:** identifiedBy: Hao Jun-Xue; **Event:** year: 2023; month: 3; **Record Level:** type: specimen; language: en; collectionCode: Algae
- **Type status:** Other material. **Occurrence:** catalogNumber: SXU-SX230331; recordedBy: Liu En-Hui; occurrenceID: 5677613E-26D2-51C6-A14B-4A62A0A4A514; **Taxon:** scientificName: *Hydrurusfoetidus*; **Location:** locality: the Fenhe River, Shanxi Rrov., China; verbatimElevation: 1356.05 m; verbatimCoordinates: 38.3588 N 111.9287 E; **Identification:** identifiedBy: Hao Jun-Xue; **Event:** year: 2023; month: 3; **Record Level:** type: specimen; language: en; collectionCode: Algae

#### Description

**Figure 2.**
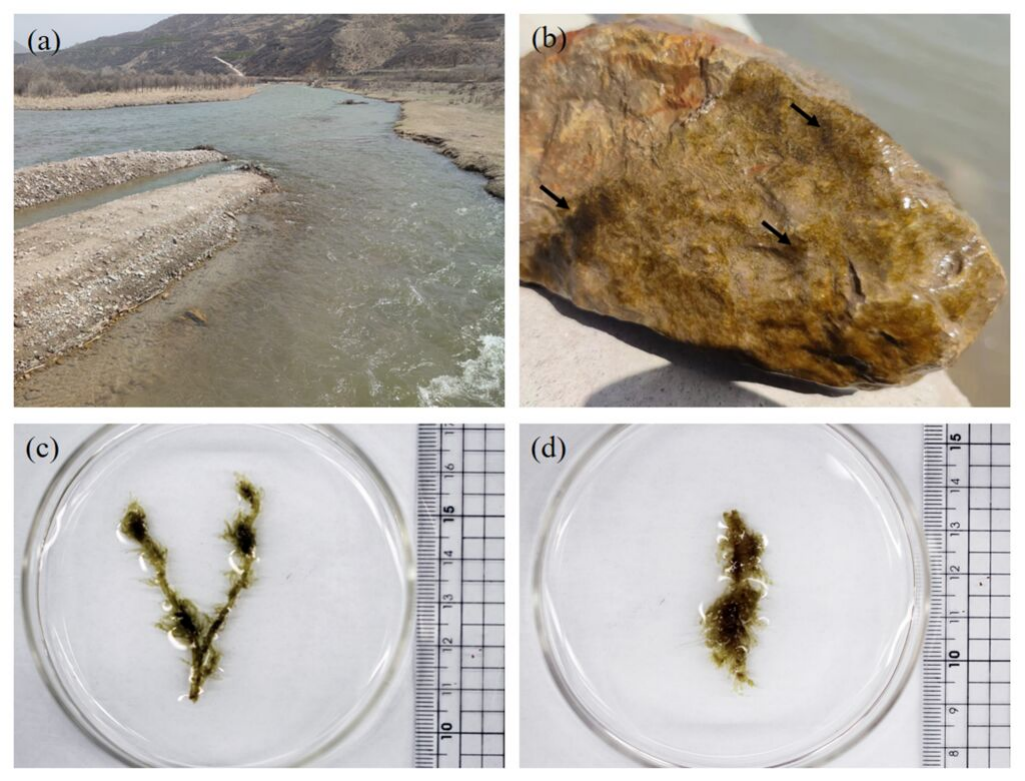
Habitat and habitus of *H.foetidus*. **a** Habitat in the Fenhe River in March 2023; **b** Colony of *H.foetidus* collected from Shanxi growing on a stone surface, the dark green surface of the stone indicating colonies, shown by the arrow; **c, d** Macroscopic morphology of *H.foetidus* collected from Shanxi.

**Figure 3.**
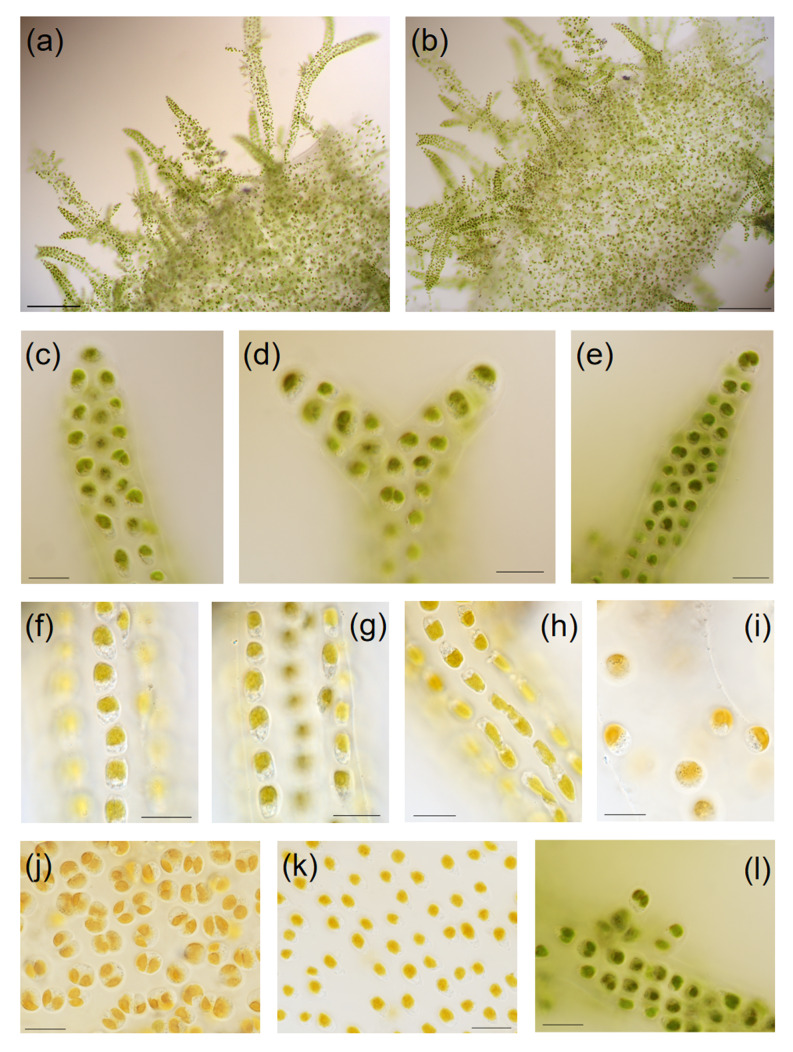
Morphological structures of *Hydrurusfoetidus* collected from Shanxi, China observed by light microscope (LM). **a, b** arbuscular thalli of *H.foetidus*; **c-e** Larger magnifications of the distal part of thalli; **f-h** Intercalary cells of dense branches, cell division was happening in **h; i** free cells of loose branches and the polysaccharide sheath were visible; **j, k** Central axis cells; **l** newly-generated apical cells. Scare bars: a, b = 200 μm, c–l = 20 μm.

The morphological characters of the specimen are shown in Figs 2, 3. The *Hydrurus* thalli, ranging from green to dark brown, were securely attached to the surface of stones, see Fig. 2a and c. The thalli collected in Shanxi were approximately 5 cm in length. The thalli partially fragmented due to river erosion. Each thallus consisted of a firm central axis and peripheral branches, encased in a viscous gelatinous coating. Macroscopic views of the specimen are depicted in Fig. 2b and d and microscopic details of specimens in Shanxi are shown in Fig. 3.

The dimensions of different cells of specimens in Shanxi were measured. The apical cells of branches, 6.00–17.14 μm × 4.52–13.57 μm, were half spheroid with flat or angular (Fig. 3c-e). The central axis cells were ellipsoid to ovoid and were 7.14–17.85 μm × 4.28–9.28 μm in size (Fig. 3j and k). The branch cells were 5.88–19.99 μm × 4.41–9.34 μm (Fig. 3f–i). The branch cells were further categorised into dense branching intercalary cells and loose branching free cells. The intercalary cells of the dense branches were ellipsoid with angular or compressed shapes, while the free cells of the loose branches were spherical to spheroid.

#### Distribution

The Fenhe River is the second largest tributary of the Yellow River, flowing through six cities in Shanxi Province. *Hydrurusfoetidus* was collected in the Xinzhou section of this river. The physical and chemical parameters of water at four sites along the Fenhe River were recorded in Table 2 and the results revealed the following: the sites where *H.foetidus* was found ranged in altitude from 1356.05 to 1869.2 m, the water temperature ranged from 4.3℃ to 9.8℃, the pH ranged from 7.04 to 7.24. The lowest total dissolved solids content was 279.5 ppm, while the highest was 604.5 ppm. The dissolved oxygen concentration peaked at 15.22 mg/l, while the lowest was 12.27 mg/l. The electrical conductivity was lowest at 429.9 μS/cm, with other sites exhibiting values around 900 μS/cm. During our visit, the watercourse of the Fenhe River was observed to be between 70 and 200 cm deep. Notably, the Secchi depth of water at the source of the Fenhe River was 200 cm, significantly deeper than the 70–80 cm depths recorded at the other three sites. Total nitrogen levels ranged from 1.165 mg/l to 2.225 mg/l and total phosphorus levels were between 0.025 mg/l and 0.055 mg/l. COD varied between 41 mg/l and 50 mg/l and ammonium levels ranged from 0.35 mg/l to 0.47 mg/l.

## Analysis

### Phylogenetic analysis

**Figure 4.**
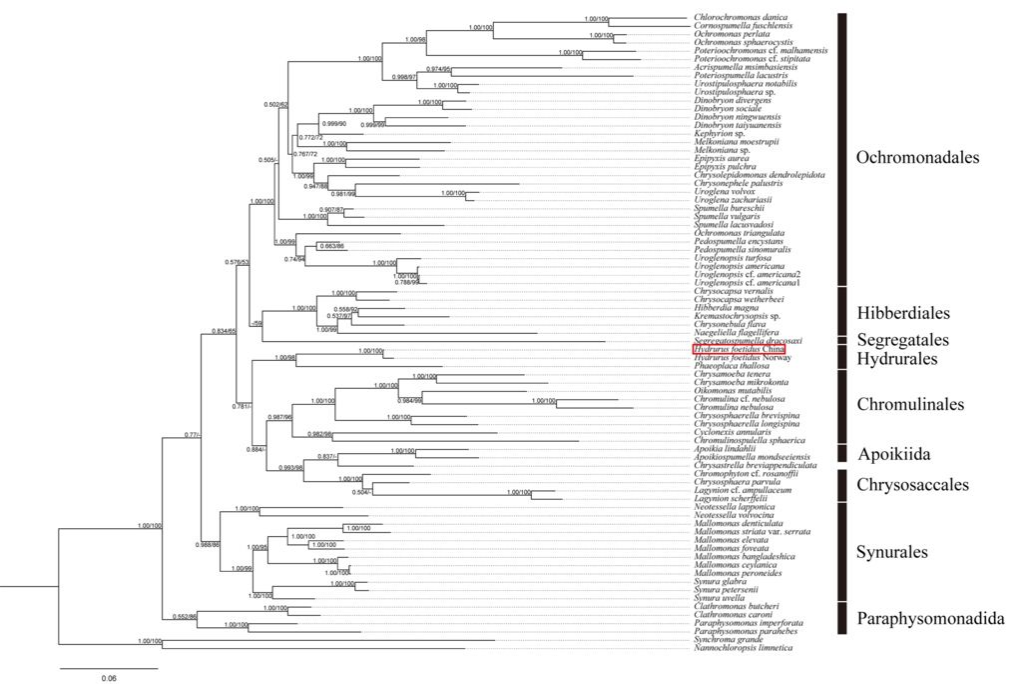
Bayesian Inference (BI) tree, based on concatenated SSU, LSU and *rbc*L rDNA sequences. Support values > 50% for all analyses are shown as follows: Bayesian posterior probabilities (BI)/Maximum Likelihood bootstrap values (ML). ‘-’ denotes < 50% support for that analysis at that node. Red boxes indicate the *Hydrurus* specimens collected from Shanxi Province, China.

The molecular phylogeny was conducted, based on SSU, LSU and *rbc*L rDNA to reveal the placement of *Hydrurus* within Chrysophyceae. Pairwise distances, based on three genes, are respectively listed in Suppl. materials 2, 3, 4. The tree topologies, based on both methods including Maximum Likelihood and Bayesian Inference, were similar. Thus, only the BI trees containing all of the supporting values are shown in Fig. 4. In the phylogeny, two strains of the genus *Hydrurus* were grouped into a single clade, which was sister to *Phaeoplacathallosa* with strong support (1.00/98). Within the monophyletic clade of *Hydrurus*, the specimen in this study clustered closely with *H.foetidus* collected from Norway supported by full values (1.00/100). Furthermore, the pairwise distance (0.0043) and base difference (21 bp) between the two *H.foetidus* strains underscored their genetic similarity. Additionally, phylogenetic relationships between *Hydrurus* and other genera of Chrysophyceae were also revealed. The clade of Paraphysomonadales diverged at the base of the phylogenetic tree (1.00/100). Within Synurales, the genus *Neotessella* was closely related to the genera *Synura* and *Mallomonas* (0.988/86). *Lagynion* was closely related to *Chrysophaera* and *Chromophyton* with high supporting values (1.00/100). *Apoikia* was clustered together with the genus *Apoikiospumella* (1.00/100). Within Chromulinales, *Chrysamoeba* was closely related to *Oikomonas* and *Chromulina* (1.00/100). *Chrysosphaerellabrevispina* and *C.longispina* formed a sister group supported by high values (1.00/100). The genera *Cyclonexis* and *Chromulinospumella* diverged at the base of the clade of Chromulinales (0.982/98). *Naegeliella* and *Chrysonebula* were closely related to *Kremastochrysopsis* and *Hibberdia* (1.00/99). *Segregatospumelladracosaxi* formed an independent clade supported by low values (-/59). *Ochromonas* was not monophyletic, *Ochromonasperlata* and *O.sphaerocystis* were closely related to the genera *Chlorochromonas* and *Cornospumella* (1.00/100), while *O.triangulata* was closely related to species of *Pedospumella* and *Uroglenopsis* (1.00/99). The genus *Spumella* was monophyletic with a high supporting value (1.00/100). *Urostipulosphaera* was closely related to *Acrispumella* and *Poteriospumella* (0.998/97). The *Dinobryon* strains shared a close relationship with *Kephyrion* sp. and *Melkoniana* species. A high supporting value (1.00/99) indicated that *Uroglena* strains were strongly connected to *Chrysonephele*, *Chrysolepidomonas* and *Epipyxis*.

### Molecular clock analyses

**Figure 5.**
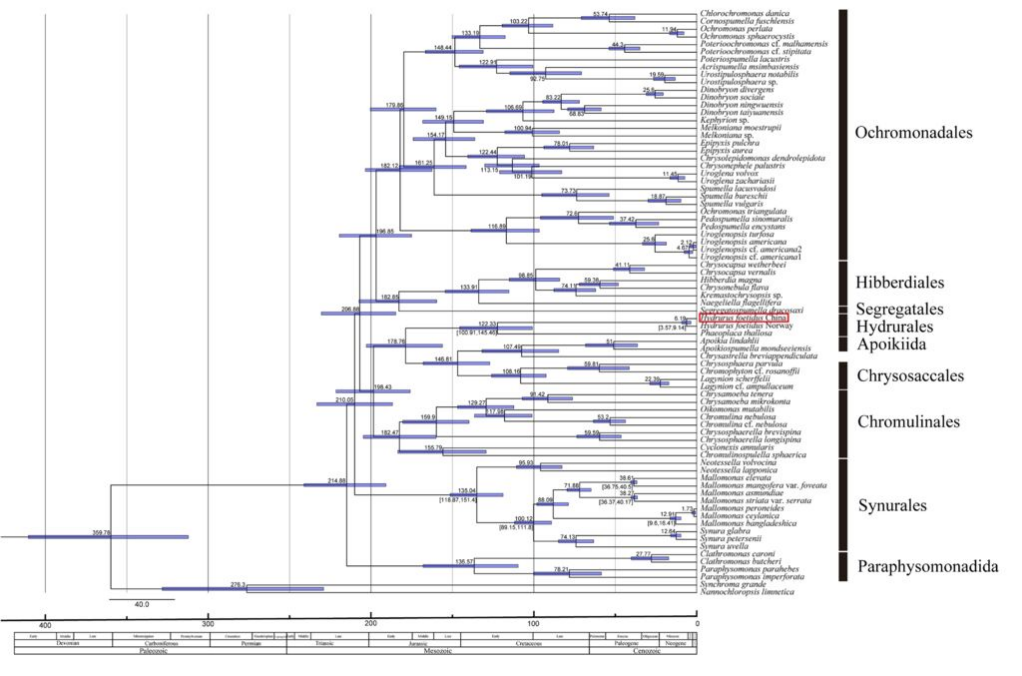
Time-calibrated phylogenetic tree from a BEAST analysis of the three-gene dataset. Values at nodes represents the mean divergence time (in million of years). Blue bars represent the 95% confidence intervals. Red boxes indicate the *Hydrurus* specimens collected from Shanxi Province, China.

Our estimates represent minimum ages primarily based on fossil remains from the Giraffe Pipe locality. Time-calibrated phylogenetic analysis estimated the origin of species within Chrysophyta (Fig. 5). Based on the Bayesian relaxed clock analyses, we estimated the origin of the genus *Hydrurus* to be in the Early Cretaceous, approximately 125 million years ago (Ma), with a likely range of 103.19 Ma to 148.86 Ma. The two *Hydrurusfoetidus* strains, collected from China and Norway, diverged between 3.7 Ma and 9.7 Ma, most probably during the Neogene period. The clade of Ochromonadales diverged from Hibberdiales and Segregatales between the Early Jurassic and Late Triassic (174.94–220.8 Ma), most likely in the Early Jurassic. Apoikiida originated approximately 107.81 Ma and Chrysosaccales originated around 148.93 Ma. The clade of Chromulinales originated between 167.93 Ma and 213.69 Ma, most likely in the Early Jurassic. The clade encompassing *Mallomonas* and *Synura* diverged from *Neotessella* between the Early Cretaceous and Late Jurassic (119.13–151.79 Ma). *Mallomonas* diverged from *Synura* between 88.92 Ma and 111.91 Ma. Paraphysomonadales originated approximately in the Late Triassic (214.59 Ma).

### ITS2 secondary structures

**Figure 6.**
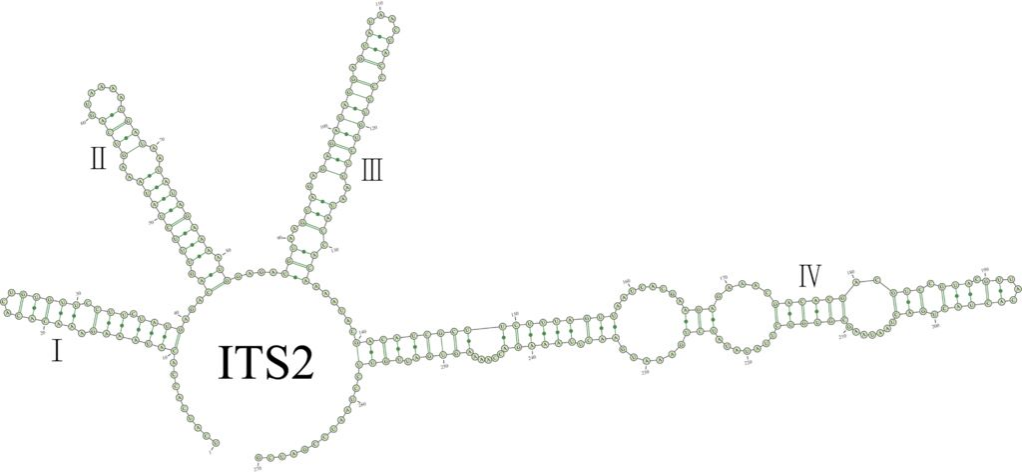
ITS2 secondary structure of *Hydrurusfoetidus*. Base numbering is indicated every 10 bases and the four helices are numbered with Roman numerals.

The ITS1-5.8S rDNA-ITS2 region of *Hydrurusfoetidus* was sequenced and the ITS2 secondary structure was constructed (Fig. 6). The ITS1 sequence length was 308 bp, the 5.8S sequence was 154 bp and the ITS2 sequence was 270 bp. Within the species of *H.foetidus*, a “ring-pin” model structure with four extended stems was identified. The ITS2 stems are typically maintained by base-pairing interactions amongst the four canonical Watson-Crick base pairs. The base and pairing composition of ITS2 in *H.foetidus* is shown in Table 3. In each division of ITS2, the paired region predominates over the unpaired region and the helix region is larger than the loop region. The bulge region, which was an unpaired portion of the helix region, occupies approximately a quarter of the total length (24.07%). Heterogeneity is presented in the base composition of ITS2. The base content reveals a hierarchy of A > U > C > G, with a high AU content accounting for 60.53% of the total bases, followed by GC content. A comparative analysis was conducted showing that helix I has a relatively lower purine content, being 0.88 times that of pyrimidine. In contrast, helices II–IV are dominated by purine.

## Discussion

Early reports suggested that the genus *Hydrurus* was widely distributed in the Holarctic Region, encompassing cold temperate inland localities (Ward 1994). A subsequent study by Klaveness (2019) narrowed down the distribution areas outside an approximate 40° N to 40° S belt around the equator. When studying the distributions of *Hydrurusfoetidus*, we discovered confirmed occurrences in the Patagonian Andes of Challhuaco, South America and the Enguri River in Georgia (Villanueva et al. 2010, Barinova and Kukhaleishvili 2017). However, due to the lack of observed sightings, the latitude range of the distribution of *H.foetidus* remains uncertain. Apparent exceptions to the distribution limits include a high-mountain location in east Turkey at 39°44′ N (Cevik et al. 2007), an outflow from the Lirung glacier in Nepal (Hirano 1969), a high mountain in Tibet, China (Raju and Suxena 1979), the Vakhsh River Basin lakes (Barinova et al. 2015) and the Pamir aquatic habitats (Barinova and Niyatbekov 2018). In this study, *H.foetidus* thalli was collected from Shanxi Province, China, at about 38° N, which is included in the distribution of that proposed by Klaveness (2019). Additionally, the effect of altitude on distribution should not be ignored; the species *H.foetidus* prefers freshwater habitats located at higher altitudes (Mašić et al. 2020). In addition, the four sites of *Hydrurus* specimens collected in this study were all near the source of the Fenhe River, aligning with earlier reports indicating a preference for *Hydrurus* to inhabit the source of rivers (Klaveness 2019). Consequently, we suggest expanding the latitude range of the distribution of the genus *Hydrurus* and emphasising the need to further explore the geographical diversity of this genus.

*H.foetidus* is an important indicator of clean water and good ecological status (Klaveness 2017). The species preferentially inhabits fast-flowing waters with low temperatures, exhibiting both rheophilic and psychrophilic properties (Klaveness 2017, Klaveness 2019). Several studies have revealed that the highest water temperature in the distributions of *Hydrurus* does not exceed 16℃, maintaining a pH range of 7.5–8.3 and an oxygen content range of 10.6–15.1 mg/l (Bursa 1934, Redžić 1988, Krizmanić et al. 2008, Stanković and Leitner 2016). In our study, the water temperature ranged from 4.4℃ to 6.3℃, with a pH of 7.04–7.24 and oxygen of 14.09–15.22 mg/l. We find certain similarities in the ecological characteristics of various habitats, particularly regarding water temperature, pH and oxygen levels. Our results support the reported ranges for water temperature and oxygen levels while expanding the pH range. *Hydrurus* is sensitive to temperature and other chemical properties. Therefore, *H.foetidus* prefers freshwater habitats characterised by clean, cold, flowing water sources such as springs, streams, lakes and rivers. In addition, the growth of *Hydrurus* exhibits certain seasonality. Typically, *H.foetidus* survives primarily from autumn to the subsequent spring, disintegrates and disappears in summer and re-appears in early autumn. Our specimen was collected in early spring. However, we rarely discover the species in winter, possibly due to environmental factors beyond temperature.

Each thallus of *H.foetidus* is arbuscular, composed of central axes and branches. The length of thalli can reach or exceed 30 cm under favourable conditions (Bursa 1934). The detailed morphological characteristics of this species have been described and previous studies have provided cell size of *H.foetidus*: 8–16 × 12–22 μm (Mack 1953); 7–20 μm (Joyon 1963); 5–15 μm (Vesk et al. 1984). Klaveness and Lindstrøm (2011) separated measurements of the different cell categories, including apical cells (9–16 × 12–19 μm), central axis cells (20–32 × 8–12 μm) and branch cells (9–17.5 × 10–18 μm). In this study, we measured the cell size of the wild *Hydrurus* thalli collected from Shanxi Province. The cell size in this study was smaller than those reported in 2011, possibly due to variations in growth conditions between wild and cultured algal strains. In addition, the species exhibits obvious phenotypic plasticity and the taxonomic history of the genus *Hydrurus* is complex and uncertain; therefore, relying only on morphological characteristics to study the species diversity is not sufficiently accurate.

However, the molecular sequences of the genus *Hydrurus* are scarce. Previous studies only provided three sequences and conducted phylogenetic trees to safely confirm *Hydrurus* as a chrysophyte (Lim et al. 1986, Klaveness et al. 2011). The absence of additional sequences of the genus *Hydrurus* underscores the need for more comprehensive molecular information to further determine the phylogenetic placement of the genus *Hydrurus* lineage. In our study, only the molecular sequences from the Shanxi specimen were obtained. We provided the first *rbc*L and ITS sequences for *H.foetidus* and predicted the ITS2 secondary structure of *H.foetidus*. Furthermore, phylogenetic trees were conducted, based on concatenated SSU, LSU and *rbc*L sequences to clarify the taxonomic status of *H.foetidus*. The close relationship between *Hydrurus* species collected in Shanxi, China and Norway was inferred. The results of this study complement molecular evidence for the genus *Hydrurus* and enhance our comprehension of the species diversity of chrysophyta.

Molecular clock analysis is rarely employed in the genus *Hydrurus*. Čertnerová et al. (2019) briefly alluded to the origin of the genus *Hydrurus* in their evolutionary study of *Mallomonas*. Based on our relaxed clock analysis, the genus *Hydrurus* originated in the Early Cretaceous (approximately 125 Ma). The estimate is closer to the 130 Ma origin deduced by Čertnerová et al. (2019). In addition, our estimated diversifications of Synurales are slightly younger compared to the analyses presented by Siver and Čertnerová et al. (Siver et al. 2015, Čertnerová et al. 2019). Our time-calibrated phylogenetic analysis offers a valuable reference for the evolutionary history of Chrysophyta. Of course, more fossil discoveries and their application to the study of the evolutionary history of chrysophyta are crucial for further advancements.

New geographical distribution of the genus *Hydrurus* was discovered in the Fenhe River, Shanxi, China. Both morphological characteristics and molecular phylogeny strongly supported the new record of *Hydrurusfoetidus* in China. This species exhibited temperature sensitivity and displayed distinct seasonal variations. The visible thallus consisted of a firm central axis and peripheral branches, with varying cell shapes. The phylogenetic analysis revealed a close relationship between *H.foetidus* specimens from China and Norway. Furthermore, the time-calibrated phylogenetic analysis inferred that the genus *Hydrurus* originated most likely in the Early Cretaceous. This study supplements new molecular evidence of *H.foetidus*, enriching our knowledge of the species diversity and evolutionary history of the genus *Hydrurus*.

## Supplementary Material

XML Treatment for Hydrurus foetidus

B51A5479-D6E8-5682-AA35-FD75F3AF9603 10.3897/BDJ.12.e137389.suppl1 Supplementary material 1 Table S1 Data type Table Brief description Taxa and accession numbers used in this study. Newly-acquired strain is highlighted in bold. File: oo_1133264.xlsx https://binary.pensoft.net/file/1133264 Junxue Hao, Yalu An, Fangru Nan, Junping Lv, Qi Liu, Xudong Liu, Shulian Xie and Jia Feng

7E990980-0274-5CF1-9508-77E3B38C40FA 10.3897/BDJ.12.e137389.suppl2 Supplementary material 2 Table S2 Data type Table Brief description Pairwise distance (lower-left matrix) and number of nucleotide variance (upper-right matrix) of SSU sequence amongst the taxa in this study. File: oo_1133265.xls https://binary.pensoft.net/file/1133265 Junxue Hao, Yalu An, Fangru Nan, Junping Lv, Qi Liu, Xudong Liu, Shulian Xie and Jia Feng

123B5C8E-34B4-57DB-B97E-2DE2450EA0DB 10.3897/BDJ.12.e137389.suppl3 Supplementary material 3 Table S3 Data type Table Brief description Pairwise distance (lower-left matrix) and number of nucleotide variance (upper-right matrix) of LSU sequence amongst the taxa in this study. File: oo_1133266.xls https://binary.pensoft.net/file/1133266 Junxue Hao, Yalu An, Fangru Nan, Junping Lv, Qi Liu, Xudong Liu, Shulian Xie and Jia Feng

CDD1D69B-860B-558D-A6D1-81EFA81FC972 10.3897/BDJ.12.e137389.suppl4 Supplementary material 4 Table S4 Data type Table Brief description Pairwise distance (lower-left matrix) and number of nucleotide variance (upper-right matrix) of *rbc*L sequence amongst the taxa in this study. File: oo_1133268.xls https://binary.pensoft.net/file/1133268 Junxue Hao, Yalu An, Fangru Nan, Junping Lv, Qi Liu, Xudong Liu, Shulian Xie and Jia Feng

## Figures and Tables

**Table 1.** Primers for amplifying and sequencing of the nuclear SSU, LSU and *rbc*L rDNA.

**Designation**	**Sequence (5’–3’)**	**Reference**
SSU		
16s	CCGAATTCGTCGACAACCTGGTTGATCCTGCCAGT	Medlin et al. (1988)
16f	CCCGGGATCCAAGCTTGATCCTTCTGCAGGTTCACCTAC	
LSU		
5.8SF	CGATGAAGAACGCAGCGAAATGCGAT	Riisberg et al. (2009)
LSU 4256R	GGAWTATGACTGAACGCCTCTAAGTCAGA	
28S_25F	ACCCGCTGAATTTAAGCATATA	Jo et al. (2011)
28S_861R	GTTCGATTAGTCTTTCGCCCCT	
28S_736F	CCCGAAAGATGGTGAACTC	
28S_1440R	TGCTGTTCACATGGAACCTTTC	
28S_1228F	CCTGAAAATGGATGGCGC	
28S_2160R	CCGCGCTTGGTTGAATTC	
28S_2038F	GACAAGGGGAATCCGACT	
28S_2812R	GATAGGAAGAGCCGACATCGAA	
*rbc*L		
Chryso_*rbc*L_F4	TGGACDGAYTTATTAACDGC	Pusztai and Škaloud (2019)
Chryso_*rbc*L_R7	CCWCCACCRAAYTGTARWA	
ITS		
ITS4	TCCTCCGCTTATTGATATGC	White et al. (1990)
KN1.1	CAAGGTTTCCGTAGGTGAACC	Wee et al. (2001)

**Table 2.** Specific information and physical and chemical properties of water on the localities of *Hydrurusfoetidus*.

Site	The source of the Fenhe River	Kuaitunguan	Matou Mountain Village	Jingle Wetland Park
Coordinate [E]	112.0831	112.1085	112.0191	111.9287
Coordinate [N]	38.8574	38.6605	38.5541	38.3588
Altitude [m]	1869.2	1506.89	1497.4	1356.05
WT [℃]	9.8	4.4	6.3	4.3
pH	7.24	7.1	7.24	7.04
Salinity[ppt]	0.43	0.46	0.43	0.43
DO [mg/l]	12.27	15.22	14.09	14.68
TDS [ppm]	279.5	604.5	572	572
EC [μS/cm]	429.9	928	876	878
SD [cm]	200	80	80	70
TN [mg/l]	1.165	1.945	2.16	2.225
TP [mg/l]	0.025	0.05	0.055	0.05
COD [mg/l]	50	44	41	42
NH^4+^ [mg/l]	0.405	0.385	0.47	0.35

**Table 3.** Base and pairing composition of ITS2 in *Hydrurusfoetidus*.

	A (%)	U (%)	C (%)	G (%)	Purines/ pyrimidines	CG(GC) (%)	AU(UA) (%)	GU(UG) (%)
Total	37.41	27.04	18.19	16.67	1.18	31.58	60.53	7.89
Paired region	55.77	42.95	26.28	25.64	1.18	31.58	60.53	7.89
Helix Ⅰ	33.33	30.00	23.33	13.33	0.88	33.33	66.67	0
Helix Ⅱ	47.50	30.00	7.50	15.00	1.67	21.43	71.43	7.14
Helix Ⅲ	36.17	27.66	17.02	19.15	1.24	29.41	52.94	17.65
Helix Ⅳ	34.75	27.97	19.49	17.80	1.11	36.36	57.58	6.06
